# The meta-analysis for ideal cytokines to distinguish the latent and active TB infection

**DOI:** 10.1186/s12890-020-01280-x

**Published:** 2020-09-18

**Authors:** Zhenhong Wei, Yuanting Li, Chaojun Wei, Yonghong Li, Hui Xu, Yu Wu, Yanjuan Jia, Rui Guo, Jing Jia, Xiaoming Qi, Zhenhao Li, Xiaoling Gao

**Affiliations:** The Institute of Clinical Research and Translational Medicine, Gansu Provincial Hospital, 204 Donggang West Road, Chengguan District, Lanzhou, 730000 China

**Keywords:** Cytokine, Tuberculous, Diagnosis, Meta-analysis

## Abstract

**Background:**

One forth whole-world population is infected with *Mycobacterium tuberculosis* (*Mtb*), but 90% of them are asymptotic latent infection without any symptoms but positive result in IFN-γ release assay. There is lack of ideal strategy to distinguish active tuberculosis (TB) and latent tuberculosis infection (LTBI). Some scientist had focused on a set of cytokines as biomarkers besides interferon- gamma (IFN-γ) to distinguish active TB and LTBI, but with considerable variance of results. This meta-analysis aimed to evaluate the overall discriminative ability of potential immune molecules to distinguish active TB and LTBI.

**Methods:**

PubMed, the Cochrane Library, and Web of Science databases were searched to identify studies assessing diagnostic roles of cytokines for distinguishing active TB and LTBI published up to August 2018. The quality of enrolled studies was assessed using Quality Assessment of Diagnostic Accuracy Studies-2 (QUADAS-2). The pooled diagnostic sensitivity and specificity of each cytokine was calculated by using Meta-DiSc software. Area under the summary receiver operating characteristic curve (AUC) was used to summarize the overall diagnostic performance of each biomarker.

**Results:**

Fourteen studies with 982 subjects met the inclusion criteria, including 526 active TB and 456 LTBI patients. Pooled sensitivity, specificity and AUC for discriminating between active TB and LTBI were analyzed for IL-2 (0.87, 0.61 and 0.9093), IP-10 (0.77, 0.73 and 0.8609), IL-5 (0.64, 0.75 and 0.8533), IL-13 (0.75, 0.71 and 0.8491), IFN-γ (0.67, 0.75 and 0.8031), IL-10 (0.68, 0.74 and 0.7957) and TNF-α (0.67, 0.64 and 0.7783). The heterogeneous subgroup analysis showed that cytokine detection assays, TB incidence, and stimulator with *Mtb* antigens are main influence factors for their diagnostic performance.

**Conclusions:**

The meta-analysis showed cytokine production could assist the distinction between active TB and LTBI, IL-2 with the highest overall accuracy. No single biomarker is likely to show sufficiently diagnostic performance due to limited sensitivity and specificity. Further prospective studies are needed to identify the optimal combination of biomarkers to enhanced diagnostic capacity in clinical practice.

## Background

Tuberculosis (TB) is still an urgent public health threat and a leading cause of death in spite of worldwide application of vaccination. It has been estimated that approximately a fourth of the world’s population is infected with *Mycobacterium tuberculosis (Mtb)* [1]. The majority of infected individuals are able to control the infection and remain asymptomatic, establishing a state of latent TB infection (LTBI). But approximately 5 to 10% LTBI patients develop into active TB due to reactivation and resuscitation of dormant bacilli indicating that persons with LTBI are the largest reservoir of infectious source after activation [2]. Thus, the development of rapid and accurate new diagnostic methods that can detect *Mtb* infection, especially distinguish between active TB and LTBI, is essential for intensifying the fight against TB and implementing the End TB Strategy [3, 4].

Diagnosis of TB status is challenging due to its diverse clinical forms and outcomes [2, 5]. Current active TB diagnosis relies on microbiologic detection of the pathogen, radiological imaging or clinical manifestations. Measurement of host immune responses, like the tuberculin skin test (TST) that is the intracutaneous injection of purified protein derivative (PPD) into the forearm, and interferon-gamma (IFN-γ) release assays (IGRAs) including the QuantiFERON®-TB Gold In-Tube (QFT) assay and T-SPOT.TB test, remains the common diagnosis for TB infection [6]. However, the TST bears limited specificity due to fail in identifying non-tuberculous mycobacteria (NTM) as well as Bacille Calmette-Guérin (BCG) vaccination [7, 8]. Although the T-cell-based IGRAs have higher specificity than the traditional TST, they remain relatively insensitive and considerable indetermining results especially in immunocompromised individuals and young children [9, 10]. Another significant limitation of both TST and IGRAs is unable to distinguish between active TB and LTBI, and this greatly hampers the early treatment and control of TB [11]. Consequently, an immunodiagnostic test to discriminate the infection statues is urgent required and would be a major advance for clinical care.

Mounting data showed that the numerous cytokines and chemokines played an important role in cellular immune responses to *Mtb* infection [12–14]. Only measuring IFN-γ response by IGRAs may leave out other key molecules in *Mtb* infection diagnosis [15]. Additional biomarkers have been investigated to improve clinical diagnosis of TB and assessment of disease status. Several studies proved that interleukin (IL)-2, IFN-γ-inducible protein of 10 kDa (IP-10), IL-5 and IL-10 had promising diagnostic performance for TB infection (including both active TB and LTBI) [16–19]. Importantly, some cytokines were shown potential diagnostic value in distinguishing of patients with active disease and LTBI, such as macrophage inflammatory protein (MIP)-1β [18], or tumor necrosis factor (TNF-α), IL-12 p40 and IL-17 [20]. It also suggested that combination of biomarker could be more sensitive than single markers for differentiating between the various stages of TB infection [17, 19, 21]. Although several markers have been suggested for diagnosing TB infection as well as differentiate between active TB and LTBI, each marker showed heterogeneity in specificity and sensitivity in different studies. To verify the diagnostic values of each biomarker in TB infection is critical for the economical selection of proper item for clinical practice, especially to provide better diagnosis performance in implying the combination of biomarkers.

In the light of these limitations, we present a systematic review and meta-analysis of the literature according to evidence-based highest-standard criteria on the accuracy of different biomarkers for differentiating active TB and LTBI, to determine their diagnostic performance and operational characteristics.

## Methods

The systematic review was conducted following the guidelines of the Preferred Reporting Items for Systematic Reviews and Meta-Analyses statement (PRISMA) [22] checklist.

### Literature search strategy

Medline (using PubMed as the search engine), the Cochrane Library, and Web of Science databases were searched by two independent researchers for relevant articles published up to August 2018. The following Medical Headings and/or text words were used as search terms: “*Mycobacterium tuberculosis* or tuberculosis or TB” AND “biomarker* or marker*or cytokine” AND “sensitivity or specificity or accuracy”. We also checked manually the reference lists in the original and review articles to identify additional studies.

### Study screening and selection

Candidate studies were assessed through the title and abstract checking. Then the potentially relevant studies were carefully read with the full-text to determine whether could be included or not. Disagreements were resolved by discussion between the two researchers.

Original studies were included that met the following criteria: (1) Original studies were assessed the accuracy of cytokine levels for distinguishing between active TB and LTBI; (2) The reference standards were clearly described and each individual were diagnosed by using the reference tests; (3) Sufficient data were used to calculate the true positive (TP), false positive (FP), true negative (TN) and false negative (FN); (4) The studies were published in English. Conference proceedings, review articles, letters to the editor were excluded.

### Data extraction and quality assessment

The following data were extracted from the finally included studies: author, country, publication year, diagnostic standard, HIV status, test methods, sensitivity and specificity. For each study, 2 by 2 tables showing rates of TP, FP, FN and TN. The quality of included studies was evaluated by two researchers using the Quality Assessment of Diagnostic Accuracy Studies-2 (QUADAS-2) tool [23]. Disagreements were resolved by consensus. A study that had no domain with a high risk of bias and no domain with high applicability concerns was regarded as a high-quality study.

### Statistical analysis

Standard methods recommended for the diagnostic accuracy of meta-analyses were used [24]. The following measures of test accuracy were calculated each individual study: sensitivity, specificity, diagnostic odds ratio (DOR), together with 95% confidence intervals (CIs). Summary receiver operating characteristic (SROC) curve was constructed for each cytokine in each study. Overall diagnostic performance of that cytokine was assessed as the area under the curve (AUC) [25].

Heterogeneity between included studies was evaluated with the Chi-squared test and Inconsistency (I-squared) statistic, *p* < 0.01 or I^2^ > 50% indicated significant heterogeneity, which was further analyzed through meta-analysis. We chose the appropriate statistical analysis model (random-effects model or fixed-effects model) for meta-analysis according to the result of heterogeneity analysis [26]. If there were enough studies, subgroup analysis was used to analyze potential heterogeneity between studies for a specific cytokine.

The potential publication bias of included studies was assessed by Deeks’s funnel plot [27]. All statistical tests were two-sided, with *p* < 0.05 taken as the threshold of statistical significance. Data were analyzed by using the software of STATA 12 (StataCorp, College Station, TX, USA) and Meta-DiSc software (version 1.4).

## Results

**Fig. 1:**
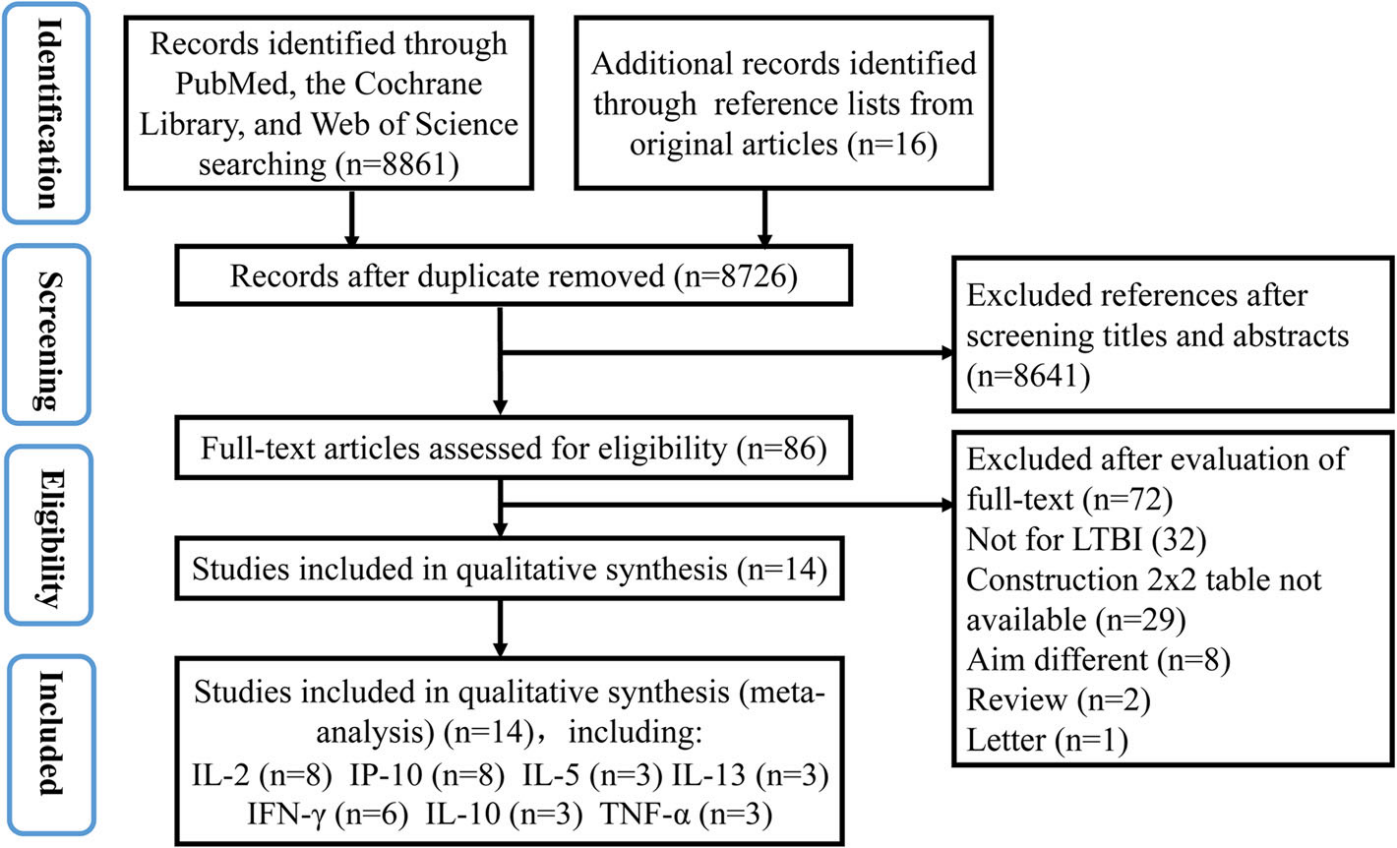
The study search and selection flow chart

After database searching and selection criterial, our systematic review and meta-analysis enrolled 14 studies examining the ability of cytokine production to distinguish between active TB and LTBI [16, 28–40]. Specifically, 8 studies with 11 independent data detected IL-2 levels [29, 31–34, 38–40], 8 studies for IP-10 representing 10 independent data [28, 30, 35–40], 6 studies for IFN-γ representing 8 independent data [28, 29, 33, 38–40], 3 studies for IL-13 representing 4 independent data [28, 29, 38]. The detection of IL-5, IL-10 and TNF-α were available from 3 independent studies. Other cytokines were excluded for our meta-analysis because relevant data resource was less than 3. The study search and selection flow chart was shown in Fig. 1.

### Characteristics and quality of included studies

**Fig. 2:**
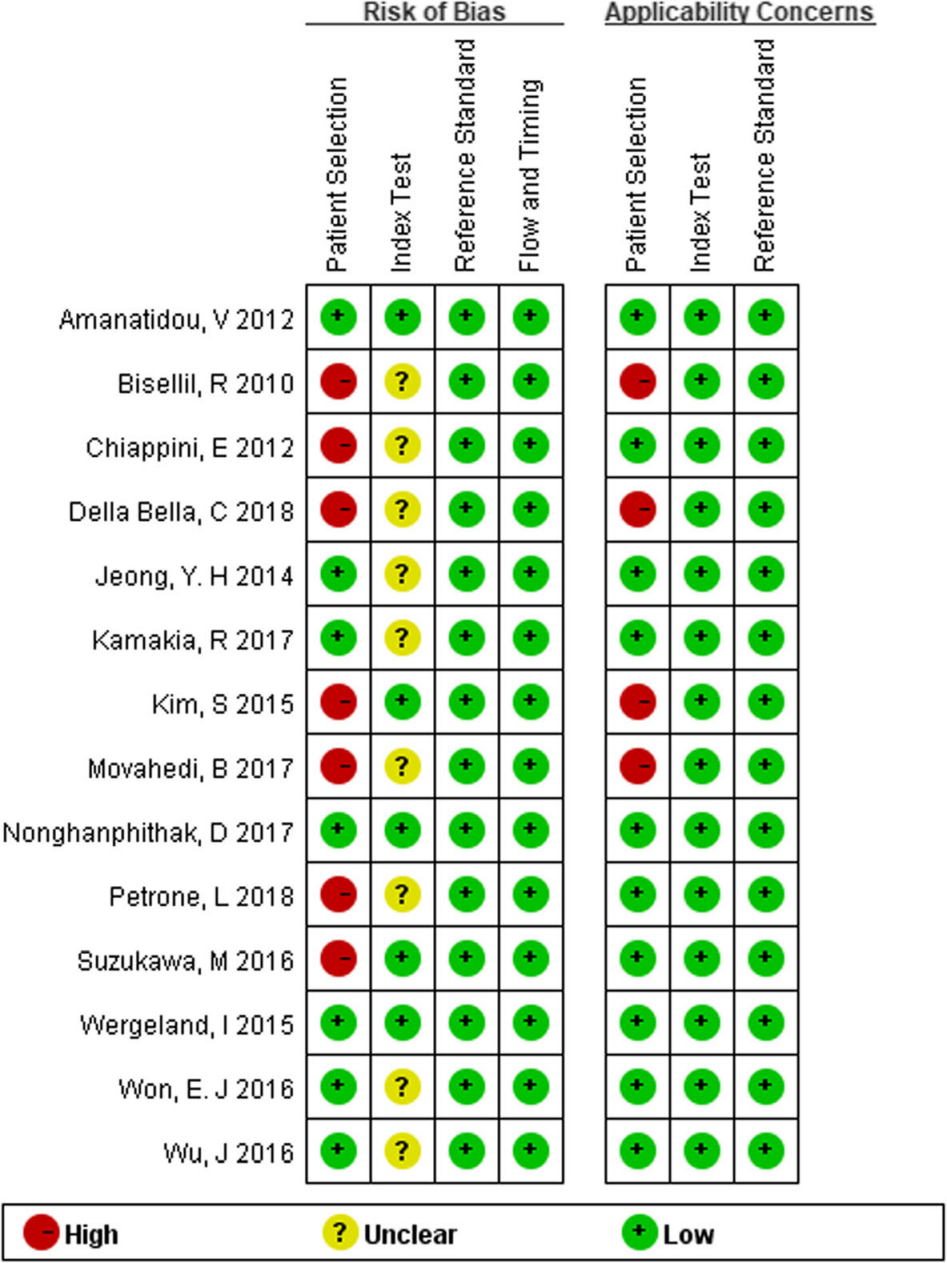
Summary of QUADAS-2 assessments of included studies. QUADAS-2: Quality Assessment of Diagnostic Accuracy Studies-2

Overall, the selected 14 studies included 982 subjects, comprising 526 active TB and 456 LTBI patients for this meta-analysis. Diagnosis of active TB and LTBI in all studies was based upon bacteriology, clinical and radiology evidence, IGRAs and/or TST, which are considered “clinical diagnosis standard”. The cytokine detection methods included enzyme-linked immunosorbent assay (ELISA), Luminex, enzyme-linked immunospot (ELISPOT) and Real Time Polymerase Chain Reaction (RT-PCR). Among these studies, 4 were performed in high TB incidence countries. The cytokine production was stimulated with different antigens, 2 studies with L-alanine dehydrogenase (AlaDH) in addition to *Mtb*-specific antigens (early secretory antigenic target-6 (ESAT-6), culture filtrate protein 10 (CFP-10), and TB7.7 antigens), 1 study with either *Mtb*-specific antigens or purified protein derivative (PPD), and the other one by AlaDH only. The rest of studies used *Mtb*-specific antigens only. The summary of included studies was shown in Table 1. Further, on the basis of patient selection, index test, reference standard, flow and timing, the risk of bias and applicability concerns were assessed by the QUADAS-2 tool. It was found that most studies had low risk of bias and an acceptable level of applicability. The result was presented in Fig. 2.

**Table 1:** The summary of included studies

Cytokines	Author (year)	Country (incidence)	Subjects (ATB/LTBI: n)	Detection methods	HIV status	Stimulator with *Mtb* antigens	TP (n)	FP (n)	FN (n)	TN (n)
**IL-2**	Suzukawa, M 2016 [38]	Japan (low)	Adults (31/29)	Luminex	negative	*Mtb*-specific antigens	30	22	1	7
	Wu, J 2016 [39]	China (high)	Adults (25/36)	Luminex	NA	PPD	21	15	4	21
	Kamakia, R 2017 [29]	Kenya (high)	Adults (19/16)	Luminex	NA	*Mtb*-specific antigens	14	1	5	15
	Movahedi, B 2017 [33]	Iranian (high)	Adults (33/33)	ELISPOT	negative	*Mtb*-specific antigens	23	19	10	14
	Movahedi, B 2017 [33]	Iranian (high)	Adults (33/33)	ELISPOT	negative	AlaDH	25	7	8	26
	Biselli, R 2010 [31]	Italy (low)	Adults (20/20)	ELISA	negative	*Mtb*-specific antigens	18	1	2	19
	Chiappini, E 2012 [32]	Italy (low)	Children (25/21)	ELISPOT	NA	AlaDH	25	4	0	17
	Della Bella, C 2018 [34]	Italy (low)	Adults (73/88)	ELISPOT	NA	ESAT-6	63	56	10	32
	Della Bella, C 2018 [34]	Italy (low)	Adults (73/88)	ELISPOT	NA	CFP-10	59	40	14	48
	Della Bella, C 2018 [34]	Italy (low)	Adults (73/88)	ELISPOT	NA	Ala-DH	70	0	3	88
	Kim, S 2015 [40]	Korea (low)	Adults (28/22)	RT-PCR	negative	*Mtb*-specific antigens	27	19	1	3
**IP-10**	Suzukawa, M 2016 [38]	Japan (low)	Adults (31/29)	Luminex	negative	*Mtb*-specific antigens	23	14	8	15
	Wu, J 2016 [39]	China (high)	Adults (25/36)	Luminex	NA	PPD	19	12	6	24
	Jeong, Y. H 2014 [28]	Korea (low)	Adults (33/20)	Luminex	NA	*Mtb*-specific antigens	23	0	10	20
	Jeong, Y. H 2014 [28]	Korea (low)	Adults (33/20)	Luminex	NA	*Mtb*-specific antigens	31	2	2	18
	Petrone, L 2018 [35]	Italy (low)	Adults (36/31)	ELISA	negative	*Mtb*-specific antigens	21	12	15	19
	Wergeland, I 2015 [36]	Norway (low)	Adults (65/34)	Luminex	positive	*Mtb*-specific antigens	65	0	0	34
	Wergeland, I 2015 [36]	Norway (low)	Adults (65/34)	Luminex	negative	*Mtb*-specific antigens	46	6	19	28
	Amanatidou, V 2012 [30]	Athens (low)	Children (54/53)	ELISA	negative	*Mtb*-specific antigens	45	11	9	42
	Nonghanphithak, D 2017 [37]	Thailand (high)	Adults (48/38)	ELISA	negative	*Mtb*-specific antigens	20	11	28	27
	Kim, S 2015 [40]	Korea (low)	Adults (28/22)	RT-PCR	negative	*Mtb*-specific antigens	28	18	0	4
**IL-5**	Suzukawa, M 2016 [38]	Japan (low)	Adults (31/29)	Luminex	negative	*Mtb*-specific antigens	27	14	4	15
	Kamakia, R 2017 [29]	Kenya (high)	Adults (19/16)	Luminex	negative	*Mtb*-specific antigens	14	1	5	15
	Won, E. J 2016 [16]	Korea (low)	Adults(36/15)	Luminex	negative	*Mtb*-specific antigens	14	0	22	15
**IL-13**	Suzukawa, M 2016 [38]	Japan (low)	Adults (31/29)	Luminex	negative	*Mtb*-specific antigens	17	13	14	16
	Jeong, Y. H 2014 [28]	Korea (low)	Adults (33/20)	Luminex	NA	*Mtb*-specific antigens	33	8	0	12
	Jeong, Y. H 2014 [28]	Korea (low)	Adults (33/20)	Luminex	NA	*Mtb*-specific antigens	26	3	7	17
	Kamakia, R 2017 [29]	Kenya (high)	Adults (19/16)	Luminex	negative	*Mtb*-specific antigens	11	1	8	15
**IFN-γ**	Suzukawa, M 2016 [38]	Japan (low)	Adults (31/29)	ELISA	negative	*Mtb*-specific antigens	28	12	3	17
	Wu, J 2016 [39]	China (high)	Adults (25/36)	ELISPOT	NA	*Mtb*-specific antigens	13	4	12	23
	Jeong, Y. H 2014 [28]	Korea (low)	Adults (33/20)	ELISA	NA	*Mtb*-specific antigens	31	8	2	12
	Jeong, Y. H 2014 [28]	Korea (low)	Adults (33/20)	ELISA	NA	*Mtb*-specific antigens	29	5	4	15
	Kamakia, R 2017 [29]	Kenya (high)	Adults (19/16)	ELISA	NA	*Mtb*-specific antigens	10	1	9	15
	Movahedi, B 2017 [33]	Iranian (high)	Adults (33/33)	ELISPOT	negative	*Mtb*-specific antigens	11	5	22	28
	Movahedi, B 2017 [33]	Iranian (high)	Adults (33/33)	ELISPOT	negative	AlaDH	16	12	17	21
	Kim, S 2015 [40]	Korea (low)	Adults (28/22)	RT-PCR	negative	*Mtb*-specific antigens	20	6	8	16
**IL-10**	Suzukawa, M 2016 [38]	Japan (low)	Adults (31/29)	Luminex	negative	*Mtb*-specific antigens	20	3	11	26
	Wu, J 2016 [39]	China (high)	Adults (25/36)	Luminex	NA	PPD	20	15	5	21
	Won, E. J 2016 [16]	Korea (low)	Adults(36/15)	Luminex	negative	*Mtb*-specific antigens	23	3	13	12
**TNF-α**	Wu, J 2016 [39]	China (high)	Adults (25/36)	Luminex	NA	PPD	20	17	5	19
	Suzukawa, M 2016 [38]	Japan (low)	Adults (31/29)	Luminex	negative	*Mtb*-specific antigens	9	2	22	27
	Kim, S 2015 [40]	Korea (low)	Adults (28/22)	RT-PCR	negative	*Mtb*-specific antigens	27	12	1	10

*ATB* Active tuberculosis, *LTBI* Latent tuberculosis infection, *Mtb-specific antigens* Combination of 3 antigens, including early secretory antigenic target-6 (ESAT-6), culture filtrate protein 10 (CFP-10), TB7.7, *AlaDH* L-alanine dehydrogenase, *PPD* Purified protein derivative, *TP* True positive, *FP* False positive, *FN* False negative, *TN* True negative, *NA* Not available

### Meta-analysis results

**Fig. 3:**
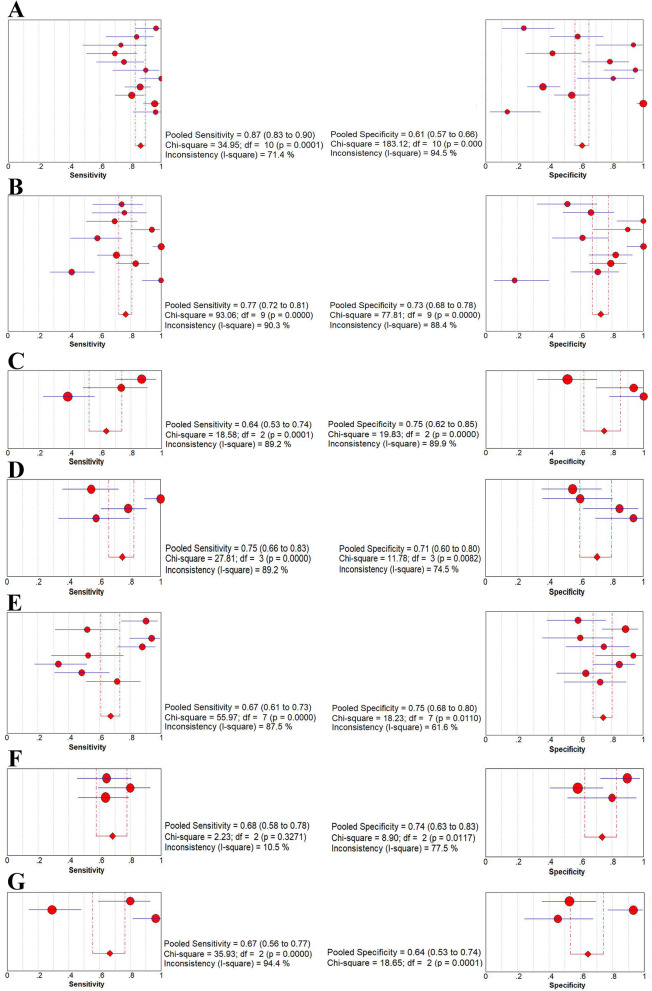
Forest plots of sensitivity and specificity of each cytokine for discriminating between active TB and LTBI. **a** IL-2, **b** IP-10, **c** IL-5, **d** IL-13, **e** IFN-γ, **f** IL-10, **g** TNF-α. The point estimates of sensitivity and specificity from each study are shown as solid circles. Error bars indicate 95% confidence intervals (CIs). Circles are proportional to study size. The pooled estimate is denoted by the diamond at the bottom

The forest plots of sensitivity and specificity for IL-2, IP-10, IL-5, IL-13, IFN-γ, IL-10 and TNF-α in discriminating between active TB and LTBI were shown in Fig. 3a-g. None of them showed less sensitivity compare to IFN-γ, even the top specificity in IFN-γ. The I-square statistic was used to detect potential heterogeneity among studies. The I^2^ values for both sensitivity and specificity were above 50% for the seven cytokines, indicating that significant heterogeneity existed among the various studies for each cytokine. It is necessary to analyze the possible interfering factors for such heterogeneity.

**Fig. 4:**
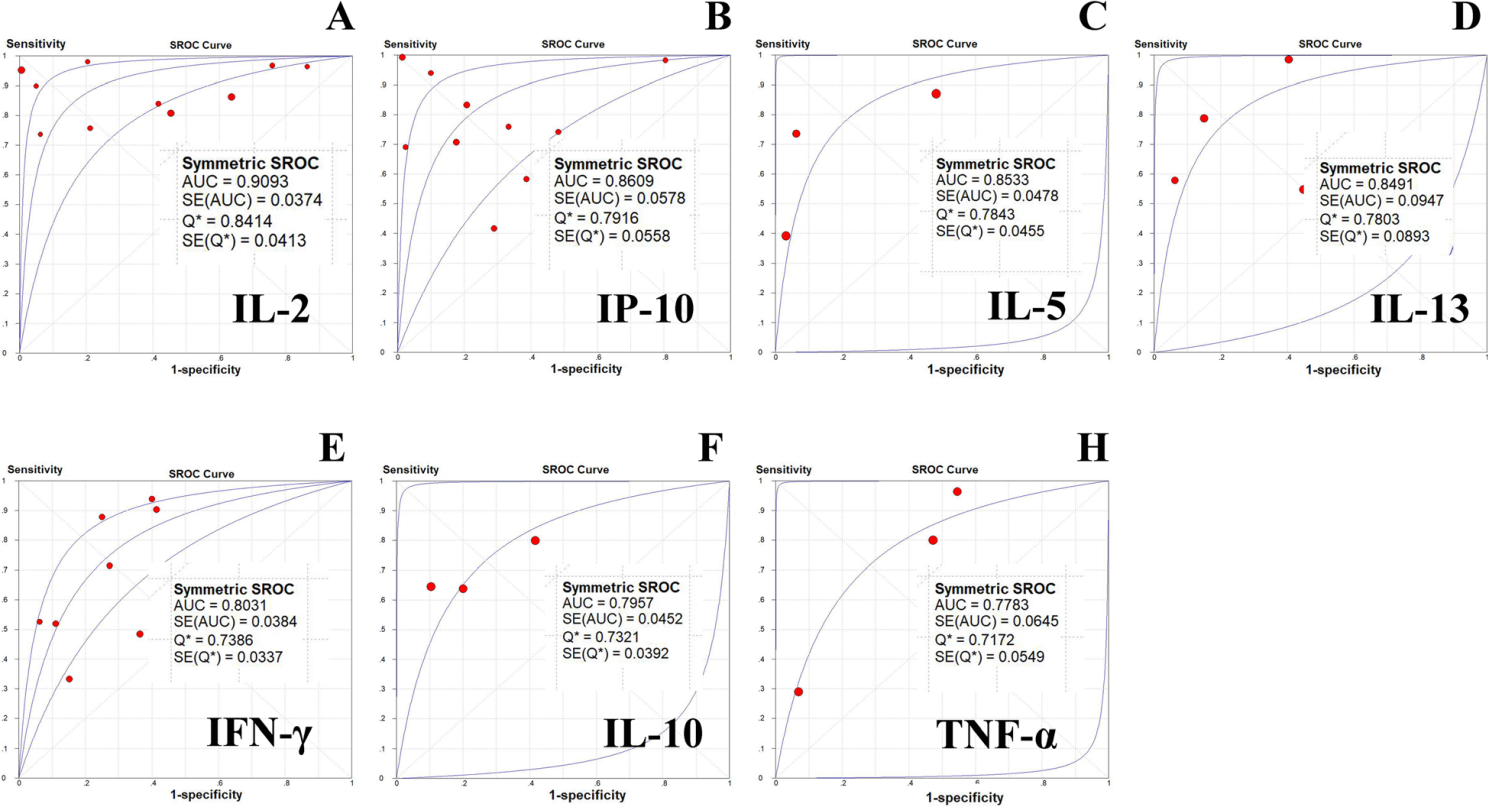
Summary Receiver Operating Characteristic curves of each cytokine for discriminating between active TB and LTBI. **a** IL-2, **b** IP-10, **c** IL-5, **d** IL-13, **e** IFN-γ, **f** IL-10, **g** TNF-α. Each solid circle represents each study in the meta-analysis. The curve is the regression line that summarizes the overall diagnostic accuracy. SROC = summary receiver operating characteristic; AUC = area under the curve; SE (AUC) = standard error of AUC; Q* = an index defined by the point on the SROC curve where the sensitivity and specificity are equal, which is the point closest to the top-left corner of the ROC space; SE (Q*) = standard error of Q* index

The diagnostic accuracy values of cytokines were assessed in a SROC curve, in which the summary operating point represents the maximum polymerization spot of sensitivity and specificity. The SROC curves for IL-2, IP-10, IL-5, IL-13, IFN-γ, IL-10 and TNF-α were present in Fig. 4a-g. The AUCs of IL-2, IP-10, IL-5, IL-13, IFN-γ, IL-10, and TNF-α were 0.9093, 0.8609, 0.8533, 0.8491, 0.8031, 0.7957 and 0.7783, respectively. Among all cytokines, IL-2 showed the highest diagnostic accuracy. IP-10, IL-5, IL-13 and IFN-γ showed an acceptable high diagnostic accuracy.

The overall diagnostic indexes of IL-2, IP-10, IL-5, IL-13, IFN-γ, IL-10 and TNF-α for discriminating between active TB and LTBI were summarized in Table 2.

**Table 2:** Pooled means of sensitivity and specificity, diagnostic odds ratio (DOR), area under the curve (AUC) for each cytokine

Cytokine	Sensitivity (95% CI)	Specificity (95% CI)	DOR (95% CI)	AUC
IL-2	0.87 (0.83–0.90)	0.61 (0.57–0.66)	13.62 (5.34–34.73)	0.9093
IP-10	0.77 (0.72–0.81)	0.73 (0.68–0.78)	12.07 (4.453–32.73)	0.8609
IL-5	0.64 (0.53–0.74)	0.75 (0.62–0.85)	11.93 (4.22–33.72)	0.8533
IL-13	0.75 (0.66–0.83)	0.71 (0.60–0.80)	12.56 (1.82–86.82)	0.8491
IFN-γ	0.67 (0.61–0.73)	0.75 (0.68–0.80)	7.44 (3.57–15.51)	0.8031
IL-10	0.68 (0.58–0.78)	0.74 (0.63–0.83)	8.20 (3.75–17.94)	0.7957
TNF-α	0.67 (0.56–0.77)	0.64 (0.53–0.74)	6.18 (2.58–14.81)	0.7783

### Subgroup analysis

To explore the factors behind the heterogeneity of sensitivity and specificity in Fig. 3, stratified (subgroup) analysis was performed using Meta-DiSc 1.4 software. In the enrolled tests, there were 11, 10 and 8 independent data for IL-2, IP-10 and IFN-γ detection, respectively. It is adequate for subgroup analysis. The rest markers under-investigated were short in the data collection which is insufficient for the subgroup analysis. So, subgroup analysis was performed for these three cytokines based on the factors of cytokine detection assays, TB incidence, and stimulator with *Mtb* antigens. As shown in Table 3, there are variabilities in sensitivity and specificity in each subgroup. When stratified by cytokine detection assays, ELISA showed better accuracy in IL-2 and higher sensitivity for IFN-γ detection (sensitivity 84%) for distinguishing between active TB and LTBI compared to other detection assays. In IP-10 detection, Luminex showed an acceptable high sensitivity (82%) and specificity (80%). However, the poor specificity of the RT-PCR assay was found in both IL-2 (14%) and IP-10 detection (18%). As well as low sensitivity of ELISPOT was found in IFN-γ detection (44%).

**Table 3:** Subgroup analysis of cytokines to distinguish between active TB and LTBI

	IL-2				IP-10				IFN-γ			
	sensitivity (95% CI)	I^**2**^ (%)	specificity (95% CI)	I^**2**^ (%)	sensitivity (95% CI)	I^**2**^ (%)	specificity (95% CI)	I^**2**^ (%)	sensitivity (95% CI)	I^**2**^ (%)	specificity (95% CI)	I^**2**^ (%)
**Detection methods**												
ELISA	0.90 (0.68–0.99)	NA	0.95 (0.75–1.00)	NA	0.62 (0.54–0.70)	90.0	0.72 (0.63–0.80)	36.2	0.84 (0.77–0.91)	19.2	0.69 (0.58–0.79)	64.5
ELISPOT	0.85 (0.81–0.89)	80.1	0.64 (0.59–0.69)	95.9	NA	NA	NA	NA	0.44 (0.34–0.55)	19.2	0.79 (0.70–0.87)	72.5
Luminex	0.87 (0.77–0.93)	67.6	0.53 (0.42–0.64)	91.5	0.82 (0.77–0.87)	87.3	0.80 (0.74–0.86)	87.7	NA	NA	NA	NA
RT-PCR	0.96 (0.82–1.00)	NA	0.14 (0.03–0.35)	NA	1.00 (0.88–1.00)	NA	0.18 (0.05–0.40)	NA	0.71 (0.51–0.87)	NA	0.73 (0.50–0.89)	NA
**TB incidence**												
High	0.75 (0.66–0.83)	0	0.64 (0.55–0.73)	83.5	0.53 (0.41–0.65)	87.7	0.69 (0.57–0.79)	0	0.45 (0.36–0.55)	0	0.81 (0.73–0.88)	68.7
Low	0.90 (0.87–0.93)	68.5	0.60 (0.55–0.65)	95.4	0.82 (0.77–0.86)	88.5	0.74 (0.68–0.79)	90.9	0.86 (0.79–0.92)	55.4	0.66 (0.55–0.76)	0
**Stimulator of** ***Mtb*** **antigens**												
*Mtb* -specific antigens	0.84 (0.8–0.89)	63.9	0.47 (0.41–0.52)	90.4	0.77 (0.72–0.81)	91.4	0.74 (0.68–0.79)	89.6	0.70 (0.63–0.77)	88.0	0.77 (0.70–0.83)	62.2
AlaDH	0.92 (0.85–0.96)	85.7	0.92 (0.87–0.96)	91.2	NA	NA	NA	NA	0.48 (0.31–0.66)	NA	0.64 (0.45–0.80)	NA
PPD	0.84 (0.64–0.95)	NA	0.58 (0.41–0.74)	NA	0.76 (0.55–0.91)	NA	0.67 (0.49–0.81)	NA	NA	NA	NA	NA

*Mtb-specific antigens* Early secretory antigenic target-6 (ESAT-6), culture filtrate protein 10 (CFP-10), TB7.7; *AlaDH* L-alanine dehydrogenase, *PPD* Purified protein derivative, *NA* Not available

Considering the population with different incidence of tuberculosis, we performed stratified basing on TB incidence. IP-10 and IFN-γ detection were less sensitive for distinguishing between active TB and LTBI in areas with high incidence of tuberculosis. However, IFN-γ detection showed high specificity (81%) in areas with high TB incidence. Compared with the high prevalence area of TB, the detection sensitivity of IL-2, IP-10 and IFN-γin the low prevalence area of TB was higher (90% vs 75, 82% vs 53, and 86% vs 45%, respectively). Moreover, when stratified by stimulator of *Mtb* antigens, AlaDH antigen showed the better accuracy in IL-2 detection for distinguishing between active TB and LTBI (sensitivity of 92%, specificity of 92%) compared to *Mtb* -specific antigens and PPD. However, IFN-γ detection was found poor sensitivity and specificity with response to AlaDH antigen, as well as acceptable sensitivity and specificity with response to *Mtb*-specific antigens.

### Publication bias assessment

The Deeks’ test indicated no evidence of bias among the studies for any cytokines meta-analyzed (Table 4). The funnel plots also showed low risk of publication bias (Supplementary Material: Figure S1).

**Table 4:** Statistical measure of publication bias for each cytokine

Cytokines	Deeks test *p* value
IL-2	0.747
IP-10	0.708
IL-5	0.052
IL-13	0.595
IFN-γ	0.075
IL-10	0.798
TNF-α	0.128

## Discussion

There is a great need for profiling biomarkers, even biomarker panels, in addition to IFN-γto improve TB diagnosis to facilitate quick and correct treatment implementation. However, there are few studies to work on it. We identified the diagnostic performance of each cytokine with the hope that our study will pave a road to certain which variables as critically essential for TB diagnosis in several settings elsewhere. In current meta-analysis, IL-2 had the highest diagnostic accuracy with total 90% AUC. And IP-10, IL-5, IL-13 and IFN-γ showed an acceptable diagnostic accuracy. Our systematic analysis data added the confidence to distinguish active TB and LTBI through fully assessment of the host immune response and combined biomarkers provided enhanced diagnostic capacity in clinical practice. To our knowledge, this is the first systematic review and meta-analysis for assessment of immune molecules’ diagnostic accuracy in the distinction of active TB and LTBI.

It is well known that Th1-type immune response and relevant cytokines play a critical protective role in the host defense against *Mtb* infection, especially IFN-γ, IL-2 and TNF-α [39, 41, 42]. However, IFN-γdetection is not ideal in our data with sensitivity of 0.67, and specificity of 0.75. In contrast, IL-2 levels had greater sensitivity, but with comparable lower specificity in the discrimination of active TB and LTBI (Fig. 3a). With their diagnosis strength, we believed that IL-2 + IFNγ combination may be an idea strategy due to the compensation of each other. Several studies have supported that IL-2/IFN-γ ratio has the potential to be a useful value to distinguish between active TB and LTBI [17, 31, 43]. The diagnostic value of the IL-2/IFN-γ ratio was based on the dynamics of functional T-cell signatures that antigen clearance are typically associated with IL-2-dominant T-cell responses, while high antigen loads are associated with IFN-γ-dominant T-cell responses [44]. The diagnostic value of the IFN-γ and IL-2 in discrimination of active TB and LTBI need further investigation. We proposed that a panel with additional molecules might be optimal besides the combination of IFN-γ and IL-2.

In our meta-analysis, other biomarkers were also evaluated for their sensitivity and specificity in distinguishing between active TB and LTBI. IL-10 can suppress T-cell proliferation and IFN-γproduction, which maybe initiate the activation of LTBI. Decreased IL-10 expression was found to release the suppression to Th1 immunity in active TB patients [39]. Further, in chronic mycobacterial infections, a higher proportion of IL-10^+^ CD4^+^ T cell subsets are found [39, 45]. In our analysis, IL-2 and IL-10 pattern was suggested to discriminate active TB and LTBI [39]. However, IL-10 detection was only found in 3 studies, with low sensitivity and specificity. The potential of IL-10 alone or in combination with other biomarkers for discriminating active TB and LTBI needs to be further evaluated. IP-10 is a chemokine that promotes Th1-type CD4^+^ T cells responses and IFN-γ upregulation, attracts monocytes and activated lymphocytes to inflammatory foci. Current studies reported that IP-10 contributes to the necrosis of tuberculous granulomas by recruiting the immune cells and inhibiting angiogenesis [46–48]. A number of studies have previously highlighted the diagnostic potential of IP-10 in distinguishing between active TB and LTBI [28, 35, 36]. Our data showed IP-10 identified active TB and LTBI with sensitivity of 77 and 73% specificity, indicating IP-10 has potential in differential diagnosis between TB diseases.

Previous studies have mentioned that the combination panel of fractalkine, IFN-γ, IL-4, IL-10 and TNF-α could distinguish active TB and LTBI [38, 49]. Another study found that the combination of TNF-α, IL-2 and IP-10 had the strongest diagnostic potential to differentiate active TB and LTBI [40]. These results all indicated that multiple cytokine pattern may improve the ability to detect various TB disease stages. More prospective studies are still necessary to identify the ideal combination.

Among our candidate cytokines, a few studies have been conducted on IL-5 and IL-13 detection. Based on the results obtained from our analysis, we reported that the sensitivity of two cytokines were 64 and 75%, and the specificity were 75 and 71%, respectively, in discriminating active TB and LTBI. Thus, these cytokines may also be a good candidate for differential diagnosis of active TB and LTBI.

The I^2^ test for the pooled sensitivity and specificity indicated that there is heterogeneity during the data analysis in our study. Stratified (subgroup) analysis for IL-2, IP-10 and IFN-γ based on cytokine detection assay, population with different TB incidence, stimulator with *Mtb* antigens. Surprisingly, we found that the accuracy of cytokine detection assays varied in different cytokine measurement. ELISA is good for IL-2 and IFN-γ detection, while IP-10 preferred Luminex detection with higher sensitivity and specificity. In contrast, both RT-PCR and ELISPOT did not reach the expectation regarding to the diagnostic performance in certain cytokines (Table 3). The results indicated that detection method is critical for different biomarkers in their diagnostic capacity.

Our results displayed the diagnostic value of certain cytokine varied at different area with different TB incidence. IP-10 and IFN-γ detection were less sensitive for distinguishing between active TB and LTBI in areas with high incidence of tuberculosis than low ones, even IFN-γdetection showed higher specificity. However, the distinguishing sensitivity of IL-2, IP-10 and IFN-γwere better in the low prevalence area of TB. Therefore, proper selection of cytokines or panels according to areas with different incidence of tuberculosis is necessary in help to improve the ability to distinguish between active TB and LTBI.

The *Mtb*-antigens were used as stimulators for cytokine detection. In our subgroup analysis, our data supported that AlaDH antigen is better compared to other *Mtb*-specific antigens and PPD, especially in IL-2 production. AlaDH antigen had different modified conformation in latent and active TB [50]. Since this antigen is missing in *M. bovis* and in BCG, it is highly specific to *Mtb*. Thus *Mtb* AlaDH might be a better candidate as a stimulator in cytokine production to discriminate between active TB and LTBI. Of course, our subgroup analysis did not fully cover the variability found in cytokine assay results across studies. Other factors, such as background TB disease, technician skill and experience or ethnic background could account for the heterogeneity.

Several limitations should be considered when interpreting the results. First, our literature search was limited to published studies that had probably missed some of the conference literature. Second, subgroup analysis of IL-5, IL-13, IL-10 and TNF-α was restricted by limited original data. The third limitation was stemmed from the study design of each original study. The non-prospective study designs may impair the quality of a study for diagnostic test accuracy.

## Conclusions

In conclusion, our systematic review and meta-analysis shows that a number of *Mtb*-specific cytokine responses, including IL-2, IP-10, IL-5, IL-13, IFN-γ, IL-10 and TNF-α, allow the distinction between individuals with active TB and LTBI. Importantly, IL-2 showed the highest overall accuracy. Single cytokine is hard to achieve a sufficient diagnostic performance to be considered as a diagnostic biomarker due to limited sensitivity and specificity. Larger, prospective studies are needed to identify the optimal combinations of cytokines before confirming the clinical utility of them as diagnostic markers to differentiate active TB and LTBI. Our findings can further help to elucidate the differences in pathogenesis and immunology between active and latent infections.

## Supplementary information

**Additional file 1: Figure S1.** The Deeks’ funnel plots for the assessment of potential publication bias in each interleukin. The plot shows the symmetric distribution of the log of diagnostic odds ratios against the inverse root of effective sample sizes (ESS), indicating the absence of any publication bias.

## Data Availability

The dataset supporting the conclusions of this article is included within the article’s additional file.

## Abbreviations

Mtb: *Mycobacterium tuberculosis*
TB: Tuberculosis
LTBI: Latent tuberculosis infection
IFN-γ: Interferon- gamma
QUADAS-2: Quality Assessment of Diagnostic Accuracy Studies-2
AUC: Area under the summary receiver operating characteristic curve
TST: Tuberculin skin test
PPD: Purified protein derivative
IGRAs: Interferon-gamma (IFN-γ) release assays
QFT: QuantiFERON®-TB Gold In-Tube
BCG: Bacille Calmette-Guérin
IP-10: IFN-γ-inducible protein of 10 kDa
GM-CSF: Granulocyte-macrophage colony stimulating factor
VEGF: Vascular endothelial growth fac
EGF: Epidermal growth factor
sCD40L: Soluble CD40 ligand
ELISA: Enzyme-linked immunosorbent assay
ELISPOT: Enzyme-linked immunospot
CI: Confidence interval
SROC: Summary receiver operating characteristic
