# Supplementary figures and images for: The meta-analysis for ideal cytokines to distinguish the latent and active TB infection
Source: BMC Pulm Med. 2020 Sep 18;20:248. doi: 10.1186/s12890-020-01280-x (PMC7502022; doi:10.1186/s12890-020-01280-x)

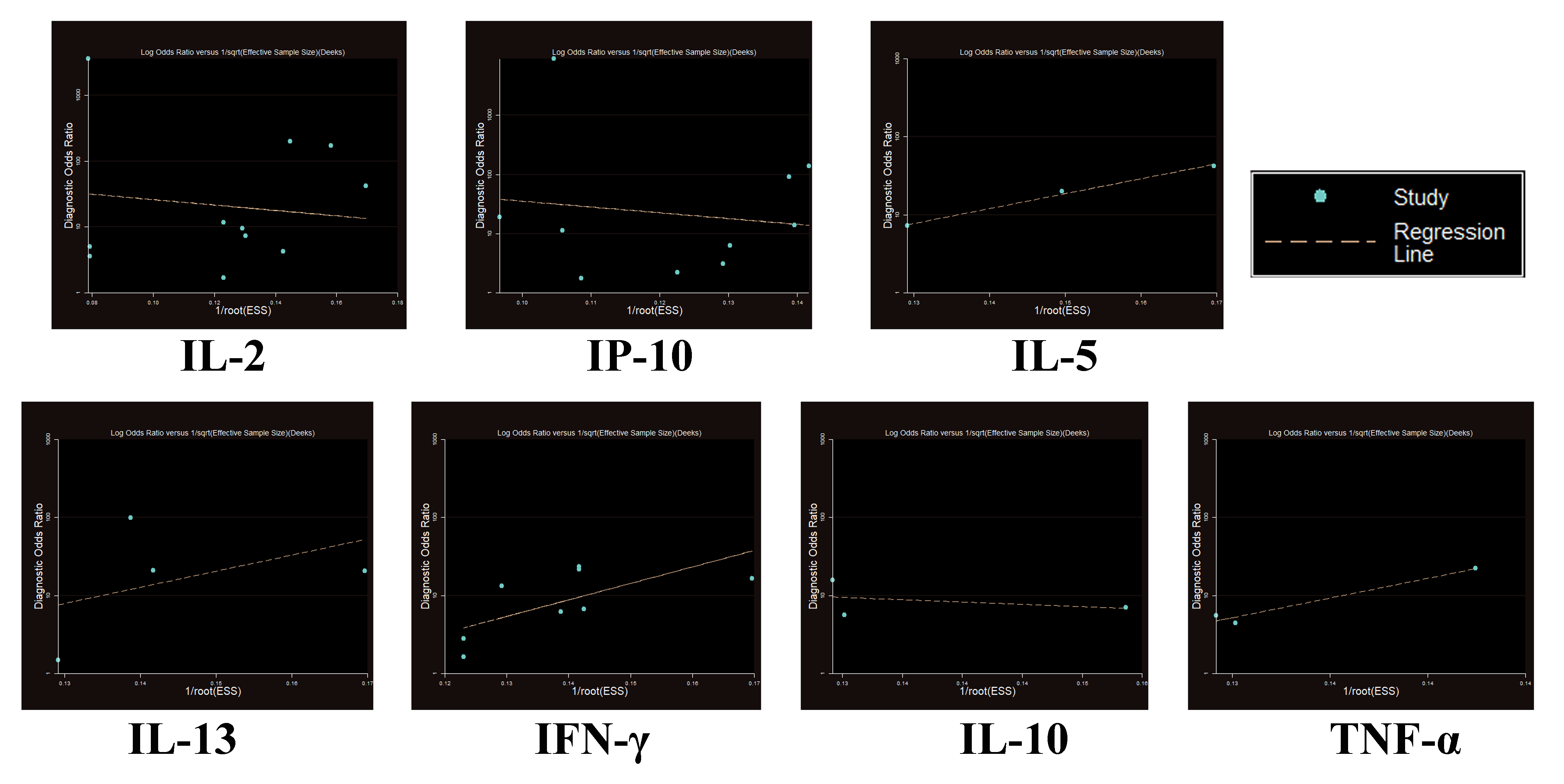

Supplement: Supplementary file 1 — Additional file 1: Figure S1. The Deeks’ funnel plots for the assessment of potential publication bias in each interleukin. The plot shows the symmetric distribution of the log of diagnostic odds ratios against the inverse root of effective sample sizes (ESS), indicating the absence of any publication bias. [file 12890_2020_1280_MOESM1_ESM.tif]
